# Ultrasound-Guided Transversus Abdominis Plane Block Versus Single-Shot Epidural Block for Postoperative Analgesia in Patients Undergoing Inguinal Hernia Surgery

**DOI:** 10.7759/cureus.33876

**Published:** 2023-01-17

**Authors:** Urvashi Yadav, Deepika Doneria, Varsha Gupta, Shipra Verma

**Affiliations:** 1 Anaesthesiology, Uttar Pradesh University of Medical Sciences, Etawah, IND

**Keywords:** postoperative analgesia, transversus abdominis plane block, ultrasound, epidural analgesia, inguinal hernia surgery

## Abstract

Background

Transversus abdominis plane (TAP) block and epidural analgesia are two frequently used regional anaesthesia techniques that attenuate postoperative pain after inguinal hernia repair.

Aim

To compare the analgesic efficacy between the single-shot epidural block and TAP block for postoperative analgesia in patients undergoing inguinal hernia repair surgery.

Methods

Forty patients of either gender undergoing elective inguinal hernia surgery of American Society of Anesthesiologists (ASA) class Ⅰ and Ⅱ were randomly allocated into two groups. Group E received a single-shot epidural with 20 ml of 0.25% bupivacaine. Group T received a TAP block with 20 ml of 0.25% bupivacaine under ultrasound guidance. Postoperative pain was evaluated by the visual analog scale (VAS). Rescue analgesia was given on VAS score ≥ 4 or on-demand in the postoperative period. The primary outcome included VAS score at 15 min, 1st h, 2nd h, 6th h, 12th h, and 24th h after the block. The secondary outcome was the analgesia duration, the total rescue analgesia dose required, and the patient satisfaction level.

Results

The VAS pain scores were significantly lower in the epidural group compared to the TAP group at the 2nd, 6th, 12th, and 24th h postoperatively (p<0.0001). The mean duration of analgesia was significantly more in Group E (576.75±96.64 min) compared to Group T (276.75±105.56 min). The total analgesic consumption was seen significantly more in 24 h in Group T than in Group E. Patient satisfaction score was significantly higher with a mean value of 5.55±0.6 in group E compared to 4.75±0.72 in group T.

Conclusion

A single-shot epidural provides better postoperative pain control than a TAP block. The duration of the first analgesic demand was prolonged, with less analgesic consumption in the epidural group.

## Introduction

Postoperative pain can affect a patient's recovery and immobilization to a greater extent [1]. Acute postoperative pain comes under the nociceptive category of pain. The protective nature of nociceptive pain is present for as long as the protection of the organism is necessary; however, if left untreated, it will persist even after healing of the initial injury [2]. Adequate analgesia modifies the pathophysiological response to stress and prevents or decreases postoperative complications, thus enhancing patient recovery. The gold standard and time-tested technique to provide postoperative pain relief is epidural analgesia. It can be used as a single-shot or continuous infusion for long-term pain relief. Aside from potentially providing excellent analgesia, its use reduces exposure to other anesthetics and analgesics, decreasing side effects and shortening lengths of in-hospital stay [3]. Newly discovered peripheral blocks, such as the transversus abdominis plane (TAP) block, have gained popularity as an adjunct to general anaesthesia for postoperative analgesia. It reduces the requirement for opioids and other analgesics with the added advantage of the absence of hemodynamic instability and early mobilization [4]. Using ultrasound increases these blocks' effectiveness by delineating the anatomy better and increasing the efficacy of these blocks [5]. Therefore, in the present study, we aim to compare the analgesic efficacy between the single-shot epidural block and TAP block for postoperative analgesia in patients undergoing inguinal hernia repair surgery.

## Materials and methods

This prospective randomized, double-blinded, comparative study was conducted in the department of anaesthesia, Uttar Pradesh University of Medical Sciences, Saifai, Etawah, India, over one year after clearance from the institutional ethical committee (EC No: 81/2019-20 with Ref.No:1662/UPUMS/Dean(M)/Ethical/2020-21). Written informed consent was obtained from all patients. The sample size was forty, with a power of 80%. The formula used for sample size calculation was n=4pq.L_2, where n = required sample size, p = approximate prevalence rate for which the study is to be conducted (obtained from the previous study), q = 1-p, and L = permissible error in the estimate. Patients posted for inguinal hernia repair with American Society of Anesthesiologists (ASA) physical status class I and II [6], weighing 40-99 kg, and fully able to understand the study contents from oral and written descriptions were included in the study. Patients with localized infection at the site of the block, hypersensitivity to local anesthetics, cardio-respiratory disorders, hepatic diseases, renal diseases, coagulation disorders or on anti-coagulant medications, spinal deformities, mental retardation, and neurological disorders were excluded from the study.

**Figure 1:**
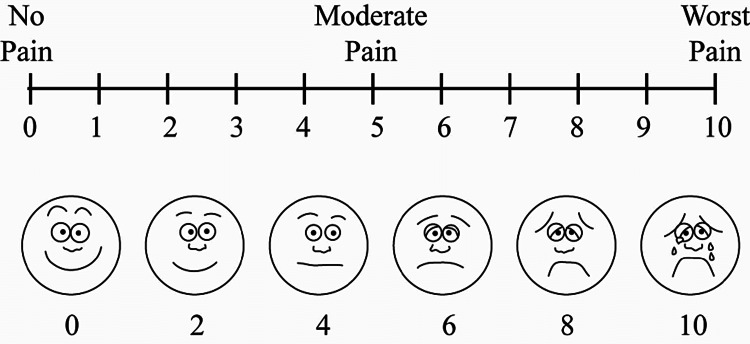
Visual analog scale.

The patients under the study were divided into two groups using a random number table, and sequentially numbered opaque sealed envelopes were prepared and randomly allocated to the patients. The patient and the observer who recorded all pain scores were not aware of the block used. All patients received instruction in the use of the VAS pain score (Figure 1), where the number 0 on the ruler represented no pain, and the number 10 represented the most severe pain ever experienced by them. They were asked to indicate the number that best reflected their pain on that scale [7].

Patients were required to fast for eight hours for solid and two hours for clear fluid before the scheduled surgery time. All patients received oral ranitidine 150 mg and oral alprazolam 0.25 mg at night before surgery. Standard monitoring comprising ECG, oxygen saturation (SpO2), and noninvasive blood pressure (NIBP) was established, and baseline hemodynamic parameters were recorded. An appropriate-size IV cannula was secured in all patients, and 10-15 ml/kg Ringer lactate infusion was started. In both groups, patients were operated on under general anaesthesia. Premedication comprised of IV injection of glycopyrrolate (5 µg/kg), injection of midazolam (0.05 mg/kg), and injection of fentanyl (2 mcg/kg). Induction was achieved by injection of propofol (2 mg/kg), and intubation was facilitated with an injection of vecuronium (0.1 mg/kg). Anaesthesia was maintained with 60% nitrous oxide in oxygen, isoflurane (MAC 1), and intermittent injection of vecuronium and fentanyl. All standard hemodynamic parameters were recorded at every 15 minutes interval till the end of surgery. After the surgery, patients were turned to a lateral position in group E just before extubation. Under aseptic precautions, a lumbar epidural Tuohy needle 18G was inserted at L3-L4 intervertebral space. After achieving 'loss of resistance to air' and negative aspiration for CSF, 20 ml of 0.25% bupivacaine was administered through an epidural needle as a single shot. Patients in group T received an ultrasound-guided TAP block at the end of the surgery, just before extubation. A portable ultrasound machine (Sonosite M Turbo) with a high-frequency linear transducer was used. Following skin and transducer preparation, the transducer was placed in the transverse plane on the midaxillary line between the subcostal margin and the iliac crest. The three muscular layers of the abdominal wall, the external oblique, the internal oblique, and the transversus abdominis muscles, were identified. The peritoneal cavity lies deep in the transversus abdominis muscle layer. A 22 gauge needle was inserted in the anterior axillary line, and the needle tip was advanced until it reached the fascial plane between the internal oblique and transversus abdominis muscles approximately in the midaxillary line. A total of 20 ml of 0.125% bupivacaine was deposited. The spread of the local anesthetic solution was visualized in real-time. After successful placement of the block in both groups, the neuromuscular blockage was reversed using an injection of neostigmine 50 mcg/kg injection of glycopyrrolate 10 mcg/kg. Patients were extubated and monitored continuously for block progression and complications.

Patients were assessed for pain and hemodynamic parameters (heart rate [HR], mean arterial pressure [MAP], SpO2, and respiratory rate [RR]) were recorded at 0 h, 15 min, 1st h, 2nd h, 6th h, 12th h, and 24th h after the block. If the patient experience pain (VAS ≥4), then an intramuscular injection, diclofenac 1.5 mg/kg, was administered. If the patient still complained of pain after 1 h of injection of diclofenac and pain score ≥4, then an IV injection of tramadol 1 mg/kg was given.

The patient's satisfaction score was assessed using a 7-point Likert verbal rating scale 24 h after the surgery as 1=extremely dissatisfied, 2=dissatisfied, 3=somewhat dissatisfied, 4=undecided, 5=somewhat satisfied, 6=satisfied, and 7=extremely satisfied [8].

Adverse events such as bradycardia (HR<60 bpm or 20% decrease from the baseline value), hypotension (fall in blood pressure by 20% from the baseline or an absolute MAP<60 mm Hg), bradypnea (RR <8 breaths per min), desaturation (<94%), nausea, vomiting, dryness of the mouth or any other events during or within the procedure were noted. Bradycardia was treated with incremental doses of IV atropine 0.3 mg. Desaturation was treated with humidified O2 inhalation. Hypotension was managed with a bolus of IV crystalloids or with increments of injection of mephentermine 6 mg.

Statistical analysis

The quantitative variables are expressed as mean ± SD and compared between groups using unpaired t-test and within groups across follow-ups using paired t-test. The Shapiro-Wilks test was used to check the normality of data distribution. Qualitative variables are compared between groups using Fisher's exact test. A p-value < 0.05 is considered statistically significant. The data is stored in an MS Excel spreadsheet, and statistical analysis is performed using IBM SPSS version 20.0.

## Results

**Figure 2:**
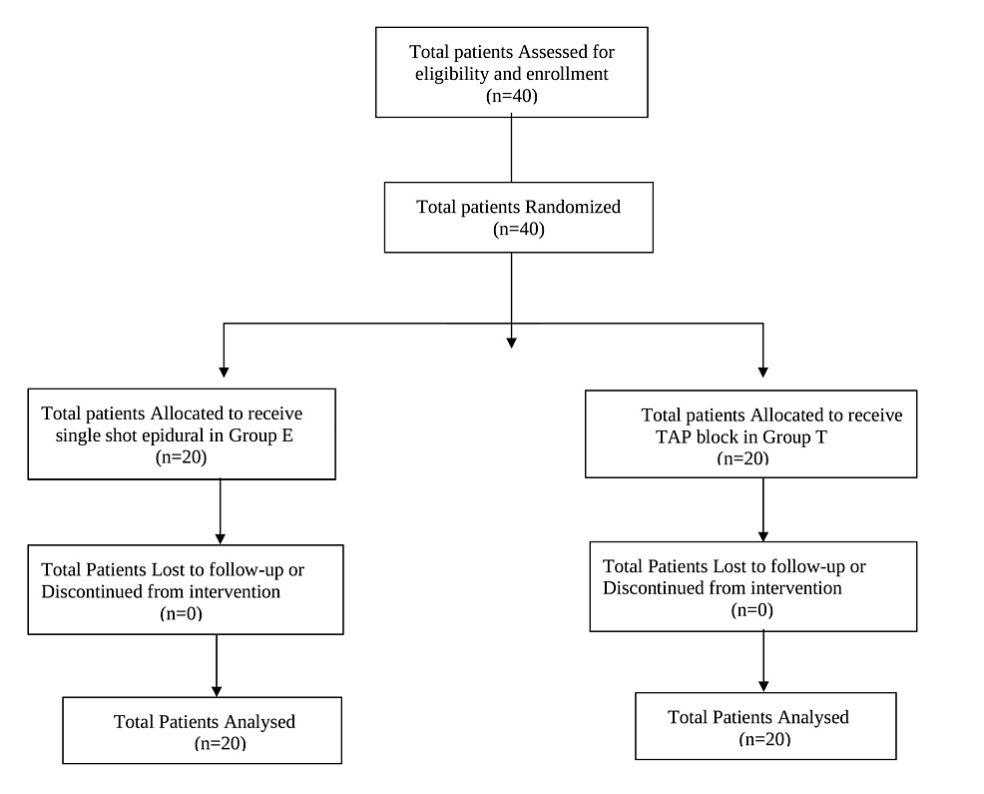
CONSORT flow diagram.

A total of forty patients were enrolled in the study, and none were excluded, as shown in the CONSORT flow diagram (Figure 2).

No significant differences were found in terms of age, height, weight, sex, duration of surgery, and intraoperative analgesic supplementation (Table 1) in both groups.

**Table 1:** Baseline characteristics of the patients. TAP: Transversus abdominis plane block; ASA: American Society of Anesthesiologists.

	Epidural		TAP		P-value
	Mean	±SD	Mean	±SD	
Age (years)	40.90	±11.63	39.35	±10.93	0.333
BMI (Kg/m^2^)	23.71	±2.25	23.38	±1.9	0.310
Duration of surgery (min)	60.00	±14.6	56.25	±13.66	0.203
Gender					
Male	18	90%	18	90%	0.500
Female	2	10%	2	10%	
ASA grade					
I	11	55%	7	35%	0.102
II	9	45%	13	65%	

The first demand for an analgesic agent was significantly earlier in the TAP group (276.75±105.56 min) as compared to the epidural group (576.75±96.64 min) (Table 2).

**Table 2:** Comparisons of VAS score in the postoperative period. *Significant (p<0.05), ^1^Unpaired t-test VAS: Visual analog scale; TAP: Transversus abdominis plane block.

Visual Analog Scale	Epidural		TAP		P-value^1^
	Mean	±SD	Mean	±SD	
15 min post-op	0.35	±0.49	0.25	±0.44	0.251
30 min post-op	0.00	±0.00	0.20	±0.41	0.018^*^
1 h post-op	0.00	±0.00	0.70	±0.80	<0.001^*^
6 h post-op	0.65	±0.67	1.60	±1.64	0.011^*^
12 h post-op	0.85	±1.18	1.90	±0.85	0.001^*^
24 h post-op	1.45	±0.83	2.50	±0.83	<0.001^*^

The total analgesic requirement was higher in the TAP group than in the epidural group. The consumption of diclofenac was significantly higher in the TAP group (150 mg±0.00) than in the epidural group (101.25±36.7 mg). Patient satisfaction score was statistically higher in the epidural (5.55±0.6) as compared to the TAP group (4.75±0.72) (Table 3).

**Table 3:** Comparison of time of first rescue analgesia (minute), rescue analgesia used, and patient satisfaction score. *Significant (p<0.05), ^1^Unpaired t-test TAP: Transversus abdominis plane block.

Parameters		Epidural		TAP		P-value^1^
		Mean	±SD	Mean	±SD	
Time of first rescue analgesia (min)		576.75	±96.64	276.75	±105.56	<0.001^*^
Rescue analgesia used	Diclofenac (mg)	101.25	±36.7	150.00	±0.00	<0.001^*^
	Tramadol (mg)	100.00	±0	136.36	±50.45	0.173
Patient satisfaction score		5.55	±0.6	4.75	±0.72	<0.001*

**Figure 3:**
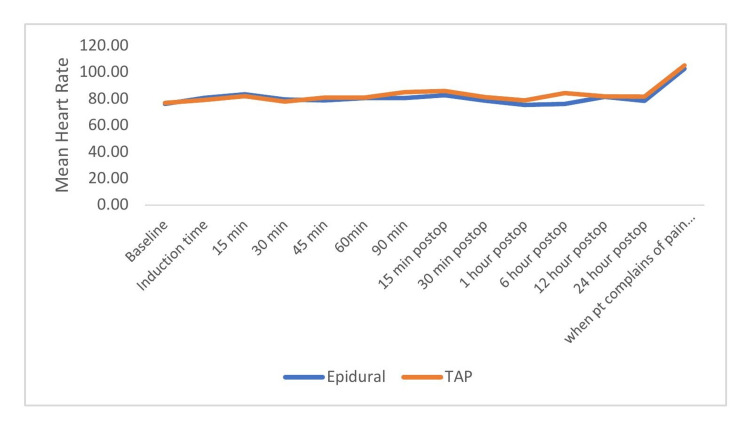
Comparison of heart rate (beats per minute) between the groups across time periods.

The heart rate was comparable during the intraoperative period but found to be significantly higher in the postoperative period after the block in the epidural group. The heart rate during rescue analgesia was increased in both groups and increased more in TAP but was statistically insignificant (Figure 3).

**Figure 4:**
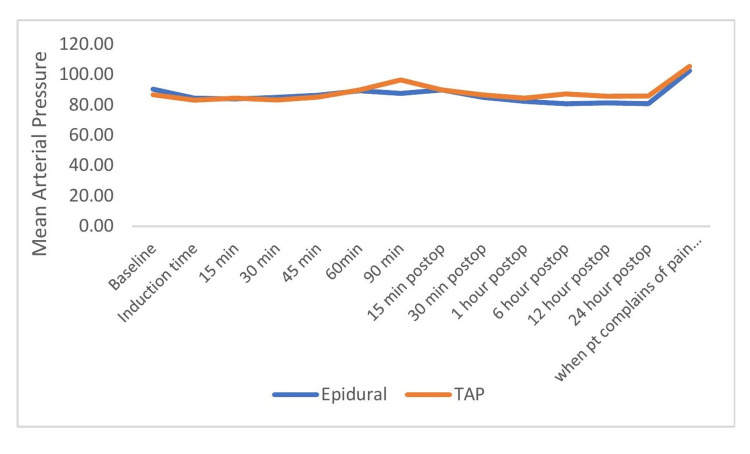
Comparison of mean arterial blood pressure (mmHg) between the groups across the time period.

The mean arterial pressure value was found to be statistically significant, being lower during the postoperative period of the 6th h, 12th h, and 24th h in the epidural group than in the TAP group (Figure 4). Changes in RR and SpO2 are comparable in both groups.

## Discussion

With a rising number of surgeries, there are efforts to study various available options that aim to reduce postoperative pain in an attempt to hasten functional recovery and minimize opioid-related systemic side effects. Regional nerve blockade is the favored alternative nowadays. The present study compared the TAP block with a single-shot epidural for postoperative pain management. Single-shot epidural block produces effective analgesia, as well as attenuation of neurogenic contribution to inflammation. However, along with sensory, they may cause motor blockade in the lower limbs. This results in delayed ambulation and recovery. The TAP block provides reliable somatic analgesia in lower abdominal surgeries. Using ultrasound helps in the correct plane localization and accurate placement of the needle and catheter. Supporters of the technique claim superior analgesia compared to that provided by systemic opioids [9,10].

In the present study, mean VAS scores were lower in the epidural group compared to the TAP group at 30 min, 1st, 6th, 12th, and 24th hours. This is consistent with the studies done by Torgeson M et al. [11], Iyer SS et al. [12], and Tejedor A et al. [13]. A meta-analysis demonstrated that patients receiving epidural analgesia compared to TAP block had experienced less postoperative pain at rest and on movement, which is consistent with our study [14,15]. These findings could be because the epidural provides somatic and visceral analgesia, and the TAP block provides only somatic analgesia [16]. In the present study, the duration of analgesia was prolonged in the epidural group compared to the TAP group. The total diclofenac requirement was higher in the TAP group than in the epidural group. Tramadol consumption was also higher in the TAP group, but the difference was not statistically significant. Similarly, Iyer SS et al. reported that the paracetamol requirement was comparable in both groups. However, the epidural group had more patients needing nil or lower doses of paracetamol [12]. They used tramadol as a second-line analgesic, and its requirement was significantly higher in the TAP group. It was consistent with studies by Tejedor A et al. and Desai N et al. [13,15]. In the present study, the mean heart rate was comparable during the surgery but found to be significantly higher during the postoperative period after the block, i.e., 30 min, 1 h, and 6 h in the TAP group. The increase in heart rate could be due to pain caused by weaning off of the effect. An increase in heart rate was observed in the TAP block. The mean heart rate was not significantly different in the epidural block with TAP block in patients undergoing lower abdominal surgery [16]. The mean heart rate was not significantly different in TAP block with epidural analgesia (EA) in an adult after abdominal surgery [17,18].

In our study, the mean MAP was comparable during the intraoperative period but significant during the postoperative period after the block at 6th h, 12th h, and 24th h in epidural and TAP groups. This was incongruent with various studies done where mean blood pressure was not significant across any period [17,18,19]. The present study shows no significant difference in terms of complications between the groups. Similar results were found in the study by Regmi S et al. [19]. The study also demonstrated no statistical difference in the incidence of hypotension, postoperative nausea, and vomiting [19]. On the contrary, Qin C et al. showed that the incidence of hypotension was significantly higher in the epidural group [14]. This may be due to a more sympathetic blockade in the epidural group. Patient satisfaction was significantly higher in the epidural group than in the TAP group. Previous studies reported that the patient satisfaction score was not significantly different between groups [20,21]. Kandi Y observed low VAS scores and high patient satisfaction levels in the TAP block group [22]. This discrepancy from our study may be because TAP block spares motor and sensory function of the lower limbs, which allows for early ambulation in patients in the TAP group, facilitates a greater degree of postoperative care, and thereby results in high patient satisfaction levels in TAP group [22]. None of the patients had any other significant side effects for which any intervention was required.

## Conclusions

Our study demonstrated that the analgesic efficacy of a single-shot epidural was better than the TAP block, as VAS scores were lower in the epidural group. Duration of the first analgesic demand was prolonged by single-shot epidural than compared to TAP block. The total analgesic consumption was less in the epidural group than in the TAP group. A greater patient satisfaction level was demonstrated in the epidural group in comparison to the TAP group. Hence, a single-shot epidural is a better postoperative analgesic technique than a TAP block in patients undergoing inguinal hernia repair surgery.
